# Exploring the Potential of SnHPO_3_ and Ni_3.4_Sn_4_ as Anode Materials in Argyrodite-Based All-Solid-State Lithium-Ion Batteries

**DOI:** 10.3390/nano15070512

**Published:** 2025-03-28

**Authors:** Wissal Tout, Junxian Zhang, Mickael Mateos, M’hamed Oubla, Fouzia Cherkaoui El Moursli, Fermin Cuevas, Zineb Edfouf

**Affiliations:** 1 Materials and Nanomaterial for Photovoltaics and Electrochemical Storage (MANAPSE), Faculty of Sciences, Mohammed V University in Rabat, Rabat 10000, Morocco; wissal.tout@um5r.ac.ma (W.T.); m.oubla@um5r.ac.ma (M.O.); f.elmoursli@um5r.ac.ma (F.C.E.M.); z.edfouf@um5r.ac.ma (Z.E.); 2 Univ Paris-Est Creteil, CNRS, ICMPE (UMR 7182), 2 Rue Henri Dunant, F-94320 Thiais, France; junxian.zhang@cnrs.fr (J.Z.); mickael.mateos@u-pec.fr (M.M.)

**Keywords:** all solid-state lithium-ion battery, anode, conversion/alloying, solid electrolyte, chlorine argyrodite, ball milling

## Abstract

All-solid-state batteries have garnered significant attention due to their potential to exceed the energy density of conventional lithium-ion batteries, particularly when alloying-based materials or lithium metal anodes are used. However, achieving compatibility with lithium metal remains a persistent bottleneck. In this study, we shed light on the potential of SnHPO_3_ tin phosphite and Ni_3.4_Sn_4_ intermetallic as novel conversion/alloying anode materials for all-solid-state lithium batteries using Li_6_PS_5_Cl as the solid electrolyte. The two Sn-based active materials were nanostructured by ball-milling to demonstrate considerable promise for application in all-solid-state half-cells. Galvanostatic cycling at room temperature revealed electrochemical behavior based on conversion/alloying reactions akin to those observed in conventional lithium-ion batteries. Promisingly, both materials exhibited satisfying electrochemical stability, with coulombic efficiencies exceeding 97%. These findings indicate that Li_6_PS_5_Cl solid electrolyte is compatible with Sn-based alloying anodes.

## 1. Introduction

The development of high-energy density lithium-ion batteries (LiBs) presents a significant technological challenge, particularly in the context of the widespread electrification of vehicles and nomadic applications. While commercial liquid organic electrolytes exhibit high ionic conductivity, ~10 mS cm^−1^ at room temperature (RT), they present substantial safety risks that compromise the reliability of LiBs [1,2]. These risks stem primarily from the inherent flammability and volatility of these electrolytes, which can result in severe hazards such as short-circuiting, leakage, and even explosions. Additionally, the limited thermal stability of these electrolytes exacerbates the risk of thermal runaway under conditions of elevated temperatures or mechanical stress. The all-solid-state lithium-ion battery (ASSLiB) represents a promising technology to address these safety concerns, offering the potential for the use of high-capacity electrode materials along with the elimination of flammable liquid electrolytes [3]. Among the various solid electrolytes being researched, sulfide-based solid electrolytes have demonstrated outstanding ionic conductivities with wide electrochemical window stability [4]. A special interest is addressed to Li_6_PS_5_Cl, a chlorine argyrodite-type electrolyte, owing to its high ionic conductivity, ~1 mS cm^−1^ at RT. Furthermore, its application in all-solid-state lithium-ion batteries (ASSLiBs) with a range of active materials has yielded highly promising results [1,5].

One of the most crucial components of the ASSLiBs, the anode, has recently shown remarkable progress, paving the way for new possibilities in battery research. Hitherto, the development of high-capacity anode materials for ASSLiBs has largely been focused on lithium metal [6,7,8,9]. This focus is driven by lithium’s high theoretical capacity (3862 mAh g^−1^) in tandem with its lowest working potential (−3.04 V vs. SHE), which maximizes the energy density of ASSLiB [7]. However, the lithium metal anode faces significant drawbacks, including lithium deposition at the electrode/electrolyte interface, which triggers the propagation of lithium dendrites into the solid electrolyte, leading to short circuits and cell failure issues [7,8]. Moreover, the high chemical reactivity of lithium metal with the solid electrolyte usually leads to the formation of various by-products at the electrode/electrolyte interface. For example, Liang et al. reported that coupling Li_3_PS_4_ solid electrolyte and lithium metal leads to in situ decomposition of the Li_3_PS_4_, generating Li_2_S and Li_3_P, which subsequently increases interfacial resistance [10]. This undesirable reaction reduces the coulombic efficiency, shortens cycle life, and causes capacity decay, ultimately reducing the overall energy density of the ASSLiB.

These issues can be mitigated by utilizing alternative anode materials that offer not only reduced reactivity toward the solid electrolyte but also higher energy density of the battery. Given that most studies in ASSLiBs have focused on Si-based anodes [8,11,12,13,14,15,16,17,18,19], we aimed to broaden the scope by exploring the potential of Sn-based materials, an underrepresented yet promising alternative. While silicon offers high capacity, its severe volume expansion and interfacial instability pose significant challenges in ASSLiBs. In contrast, Sn-based materials provide a more balanced trade-off between capacity, mechanical stability, and compatibility with solid electrolytes. In particular, Sn anode has gained significant attention owing to its high specific capacity, reaching a theoretical value of 990 mAh g^−1^ for the formation of Li_4.4_Sn alloy at an average potential of 0.4 V vs. Li^+^/Li [20]. Within this framework, prior studies have emphasized the properties of Sn powder as an anode material for ASSLiBs [8,15,21]. For example, Miyazaki et al. synthesized the Sn-based composite anode by milling Sn commercial powder together with 80Li_2_S·20P_2_S_5_ glass solid electrolyte [15]. The composite anode delivers a reversible capacity of 600 mAh g^−1^ over 80 cycles at 0.03 C. Nonetheless, as it is widely recognized, the Sn anodes undergo considerable volume changes during lithiation (up to 250% for Sn to Li_4.4_Sn) [20], a common issue in all alloying reactions. This leads to particle agglomeration along with mechanical instability on cycling.

A prevalent strategy to circumvent this issue involves embedding capacitive Sn elements into a non-active matrix capable of accommodating volumetric changes. This matrix can be either metallic, such as intermetallic compounds, or polyanionic materials, such as phosphite compounds. For the former, in a conventional liquid LiB, the nanostructured Ni_3.4_Sn_4_ compound is reported to provide a reversible capacity of 250 mAh g^−1^ over 100 cycles at 0.2 C through a two-step lithium insertion process enabled by a conversion-alloying mechanism [22]. As for the nanostructured SnHPO_3_ tin phosphite compound, it has demonstrated a reversible capacity of 677 mAh g^−1^ over 70 cycles at 0.2 C, attributed mainly to the Li*_x_*Sn alloys [23].

To the best of our knowledge, no previous studies have yet examined the electrochemical performance of Ni_3.4_Sn_4_ intermetallic as well as SnHPO_3_ phosphite compounds in ASSLiBs. Thereby, in this work, we investigate the electrochemical performance of SnHPO_3_ and Ni_3.4_Sn_4_ as novel conversion-alloying anode materials for ASSLiBs with a one-to-one comparison with their electrochemical behavior in conventional LiB with liquid electrolyte. Leveraging the excellent mechanical ductility of argyrodite electrolytes, Li_6_PS_5_Cl chlorine argyrodite was selected as the solid electrolyte, for which its crystallographic, morphological, and electrochemical properties are investigated.

## 2. Materials and Methods

### 2.1. Synthesis of Nanostructured Tin-Based Anodes

Tin phosphite compound SnHPO_3_ was synthesized by hydrothermal route: a mixture of H_3_PO_3_ (Sigma Aldrich, Saint Louis, MO, USA, 99%), SnCl_2_·2H_2_O (Sigma-Aldrich, Saint Louis, MO, USA, 98%), and oxalic acid C_2_H_2_O_4_ (SDS company, Minchinbury, Australia, 99.5%) was prepared with a molar ratio of 10:1:2. Oxalic acid addition prevents oxidation of phosphite to phosphate. The mixture was placed into a 50 mL Teflon-lined autoclave, then sealed and heated at 180 °C for 72 h, followed by natural cooling to RT. The resulting powder was washed, dried, and subsequently milled in a planetary mill under air atmosphere for 1 h. The milling was conducted in an 80 mL jar using 7 mm diameter balls made of hardened steel and stainless steel, respectively. The ball-to-powder weight ratio was 5:1, and the mill was operated at a rotational speed of 600 rpm. It is worth noting that a planetary mill (Pulverisette 7, Fritsch, FRITSCH GmbH, Idar-Oberstein, Germany) was utilized for all milling processes of this work.

Since the chemical homogeneity domain of Ni_3+*x*_Sn_4_ lies within the range 0.08 ≤ *x* ≤ 0.6 [24], the Ni-rich compound Ni_3.4_Sn_4_ (*x* = 0.4) was targeted for synthesis. First, pristine Ni_3.4_Sn_4_ alloy was synthesized through induction melting of high-purity nickel (99.99%) and tin (99.9%) metals in a stoichiometric molar ratio. The melting process was conducted in a water-cooled copper crucible under a high-purity argon atmosphere. To ensure homogeneity, the ingot was turned over and re-melted three times. Subsequently, the ingot underwent annealing at 700 °C for 7 days under argon atmosphere within a silica tube, followed by quenching in RT water. Annealing conditions were selected based on the characteristics of the Ni-Sn phase diagram [25]. The annealed alloy was manually pulverized to a particle size of approximately 125 μm using an agate mortar. Afterward, nanostructuration of Ni_3.4_Sn_4_ was achieved by mechanical milling under argon atmosphere for 20 h, following the same protocol as for SnHPO_3_. To avoid excessive thermal heating within the jar, a 10 min break was performed after each hour of milling.

### 2.2. Preparation of Li_6_PS_5_Cl Solid Electrolyte

Chlorine argyrodite Li_6_PS_5_Cl was synthesized from Li_2_S (Sigma Aldrich, Saint Louis, MO, USA, 99.98%), P_2_S_5_ (Sigma Aldrich, Saint Louis, MO, USA, 98%), and LiCl (Sigma Aldrich, Saint Louis, MO, USA, 99.99%) reagents in stoichiometric ratio to obtain the nominal composition. The mixture was milled for 10 h with 10 min break per milling hour, under argon atmosphere, using a 45 mL jar with 10 mm balls. Both the jar and balls were made of tungsten carbide (WC). The ball-to-powder weight ratio was 39:1, and the milling speed was set to 600 rpm.

### 2.3. Materials Characterization

#### 2.3.1. Structural, Morphological, and Microstructural Characterization

For all the studied materials, X-ray diffraction (XRD) patterns were obtained in Bragg–Brentano geometry on a D8 Advance DaVinci Bruker diffractometer (Karlsruhe, Germany) equipped with LynxEye detector and Cu-K_α_ radiation (λ = 0.15418 nm). The Li_6_PS_5_Cl containing samples were prepared in an argon-filled glove box using an airtight XRD holder to prevent reaction with moisture and oxygen. The XRD patterns were analyzed by the Rietveld method using FULLPROF suite software [26]. The crystallite size was evaluated from the Lorentzian contribution to the “Thompson-Cox-Hastings pseudo-Voigt” profile shape function after considering the instrumental resolution [27].

Chemical composition of nanostructured Ni_3.4_Sn_4_ material was analyzed using inductively coupled plasma-optical emission spectrometry (ICP-OES) using Agilent 5800 device (Agilent Technologies, Santa Clara, CA, USA). Morphology of materials was examined by Scanning Electron Microscopy (EDX-SEM) using a SEM-FEG MERLIN from Zeiss (Jena, Germany). Powder was spread on a double-sided carbon tape affixed to a SEM stub. Preparation was conducted in ambient air, except for the Li_6_PS_5_Cl argyrodite-containing powders, which were prepared in an argon-filled glove box and promptly transferred to the SEM device.

Particle size distribution (PSD) of both SnHPO_3_ and Ni_3.4_Sn_4_ nanostructured samples was determined using laser diffraction (LD) with the Mastersizer 3000 particle size analyzer (Malvern Panalytical, Malvern, UK). Powder was gradually added to distilled water under ultrasonic agitation at 1500 rpm until the recommended obscuration level was achieved (<25%).

#### 2.3.2. Electrochemical Measurements

**Figure 1:**
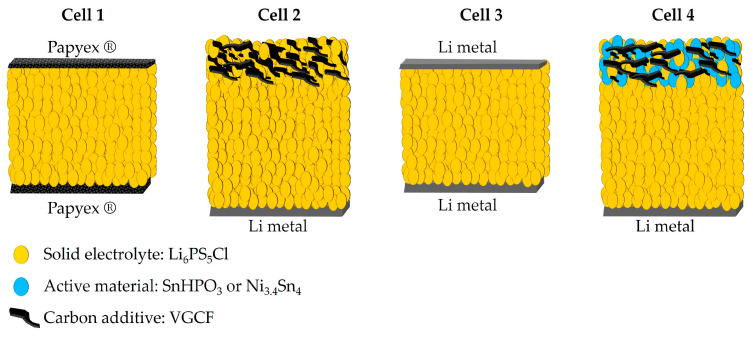
Schematic illustration of the cell configurations for the electrochemical measurements.

All electrochemical preparation processes were conducted in an argon-filled glove box (O_2_ < 4 ppm, H_2_O < 1 ppm), and all electrochemical performances were evaluated on a CR2032 coin cell using a VMP3 potentiostat (Biologic, Seyssinet-Pariset, France). Ionic conductivity of the Li_6_PS_5_Cl solid electrolyte was measured using electrochemical impedance spectroscopy technique (EIS). The solid electrolyte powder was cold pressed at 200 MPa for 2 min in a tungsten carbide die (Ø = 6 mm). The argyrodite pellet, with a thickness of 0.88 (3) mm, was then transferred to a symmetrical coin cell between two Papyex^®^ flexible graphite to ensure good electrical contact (cell 1 of Figure 1). EIS data were collected using CESH-e sample holder from Biologic (Seyssinet-Pariset, France) over a frequency range from 400 kHz to 100 Hz and with a voltage amplitude of 30 mV. Data fitting was performed using a suitable equivalent circuit model implemented in the EC-Lab V11.43 software. The activation energy (E_a_) was calculated from the Arrhenius plot of ionic conductivity recorded over the temperature range of 25 to 80 °C, using the enhanced intermediate temperature system (ITS-e) from Biologic (Seyssinet-Pariset, France).

The electrochemical stability window of Li_6_PS_5_Cl was assessed using cyclic voltammetry (CV) with the configuration depicted in Cell 2 of Figure 1. A mixture of 75 wt.% Li_6_PS_5_Cl and 25 wt.% vapor-grown carbon fibers (VGCF, VGCFTM-H, Showa Denko K.K., Tokyo, Japan) was spread on top of 38 (4) mg Li_6_PS_5_Cl pellet and then cold-pressed together under a pressure of 200 MPa. Li metal (0.25 mm thickness, 99.8%, ChemPure Chemicals, Westland, MI, USA) was attached to the other side of the Li_6_PS_5_Cl pellet. The carbon additive was added to ensure effective electronic percolation within the electrode and to mitigate the poor electronic conducting nature of the solid electrolyte. CV measurements were conducted at a scan rate of 100 µV s⁻^1^ over a voltage range from 0 to 5 V vs. Li^+^/Li.

Lithium symmetric cells were assembled using the configuration shown in Cell 3 of Figure 1 in order to evaluate the interface stability between lithium metal and Li_6_PS_5_Cl argyrodite through lithium-ion migration. The galvanostatic mode was employed at different current densities, increasing from 20 to 160 µA cm^−2^, with a time step of 30 min. In addition, a constant current density of 35 µA cm^−2^ with a time step of 4 h was conducted to evaluate the long-term interface stability.

All solid-state half-cells were assembled according to the configuration in Cell 4 of Figure 1, with lithium metal serving as both the counter and reference electrode. The composite electrode mixture (mass loading of 7 mg cm^−2^) was homogeneously distributed over the Li_6_PS_5_Cl electrolyte pellet and co-pressed together at 200 MPa for 2 min. To ensure ion percolation within both composites, the electrolyte volume fraction was fixed at 0.5. The composites were prepared by mixing active materials, Li_6_PS_5_Cl and VGCF, in an agate mortar with a weight ratio of 40:50:10 and 78:20:2 for SnHPO_3_ and Ni_3.4_Sn_4_, respectively. Carbon fibers (VGCF) have been incorporated into each electrode formulation to enhance electronic conductivity and improve lithium-ion transport within the working electrode. Half-cells were cycled in galvanostatic mode at 5 mA g^−1^ constant current within the potential range of [0.1–2] and [0.2–2] V vs. Li^+^/Li for SnHPO_3_ and Ni_3.4_Sn_4_ composites, respectively. The lower voltage cutoff was set above 0 V vs. Li^+^/Li to prevent argyrodite degradation. For comparative purposes, galvanostatic cycling was conducted for conventional lithium-ion half-cells, Li|LP30|SnHPO_3_ and Li|LP30|Ni_3.4_Sn_4_, at 108 mA g^−1^ using 1M LiPF_6_ dissolved in Ethylene Carbonate (EC)/Dimethyl Carbonate (DMC) (1:1 *v*/*v*, LP30, Sigma Aldrich, Saint Louis, MO, USA) liquid electrolyte, as described in our previous study [23].

## 3. Results

### 3.1. Structure and Morphology of Sn-Based Active Materials

#### 3.1.1. Nanostructured SnHPO_3_ Tin Phosphite

**Figure 2:**
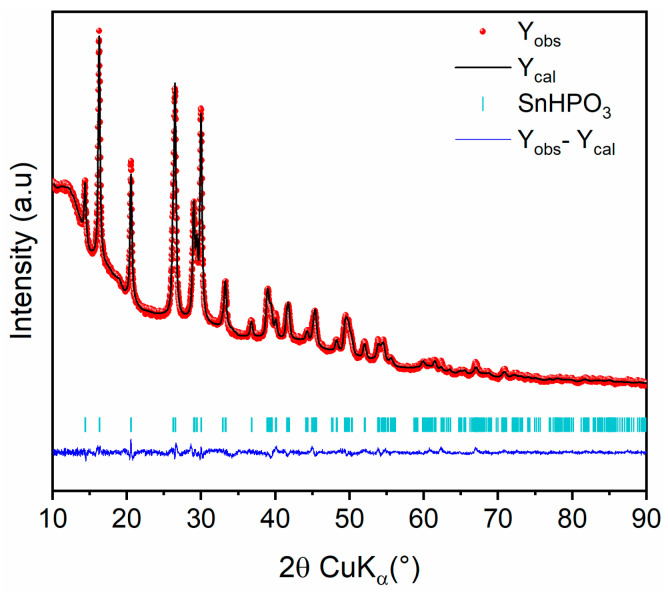
Rietveld refinement of nanostructured SnHPO_3_ XRD pattern: Observed (red dots), calculated (black line), and difference (blue line) curves are shown. The vertical marks show the Bragg positions for SnHPO_3_ phase.

The crystal structure of the nanostructured SnHPO_3_ was analyzed using XRD analysis. The Rietveld refinement of the XRD pattern is shown in Figure 2. All diffraction peaks can be indexed in a monoclinic symmetry within the *C*1*c*1 space group in agreement with the crystal structure reported by McDonald el al. [28]. The refined structural parameters are reported in Table 1. The crystallite size was evaluated from peak broadening to 24 (2) nm.

**Figure 3:**
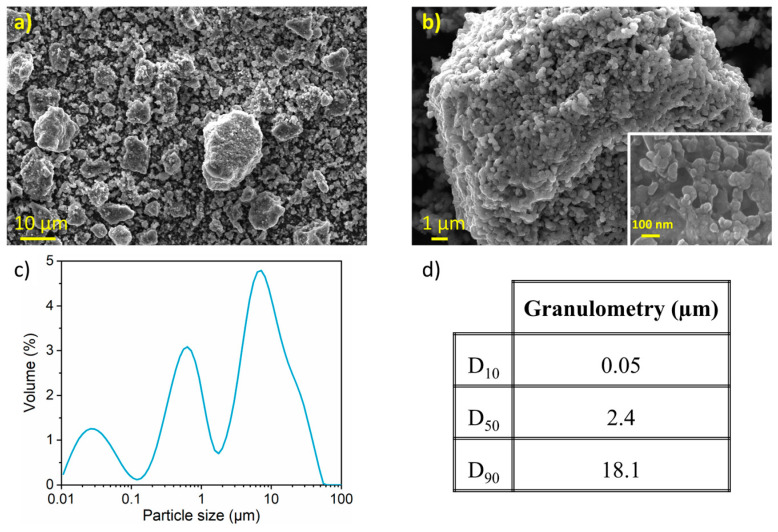
(**a**,**b**) Secondary electron image obtained with in-lens detector at three magnifications (**c**) Particle size distribution curve from LD measurements (**d**) Particle size percentiles of the nanostructured SnHPO_3_.

Figure 3 presents SEM micrographs of the nanostructured SnHPO_3_ powder. Dense and spherical secondary particles are visible, with sizes ranging from 1 to 20 μm (Figure 3a). At higher magnification (Figure 3b), granular primary particles are observed with particle size around 100 nm (inset of Figure 3b). Figure 3c shows the particle size distribution as determined by LD with its corresponding cumulative volume distribution in Figure 3d. The material exhibits a multimodal curve with a wide particle size distribution (10 nm to 60 μm) with a median particle size of 2.4 µm and a considerable extent of agglomeration.

#### 3.1.2. Nanostructured Ni_3.4_Sn_4_ Intermetallic

The crystal structure and chemical composition of the non-milled Ni_3+*x*_Sn_4_ intermetallic were analyzed using XRD and ICP analyses. Rietveld refinement of the XRD data is presented in Figure S1, and the crystallographic data are summarized in Table S1. All diffraction peaks can be indexed with monoclinic symmetry in the *C*2/*m* space group, as reported by Furuseth et al. for Ni_3+*x*_Sn_4_ phases [24]. The over-stoichiometry is accommodated through the partial occupation of the 2*a* sites, with Rietveld refinement yielding a stoichiometry of Ni_3.47(2)_Sn_4_. This result is consistent with the ICP analysis (Ni_3.46(4)_Sn_4_) displayed in Table 2. Accordingly, the composition for further study has been set to the nominal Ni_3.4_Sn_4_.

**Figure 4:**
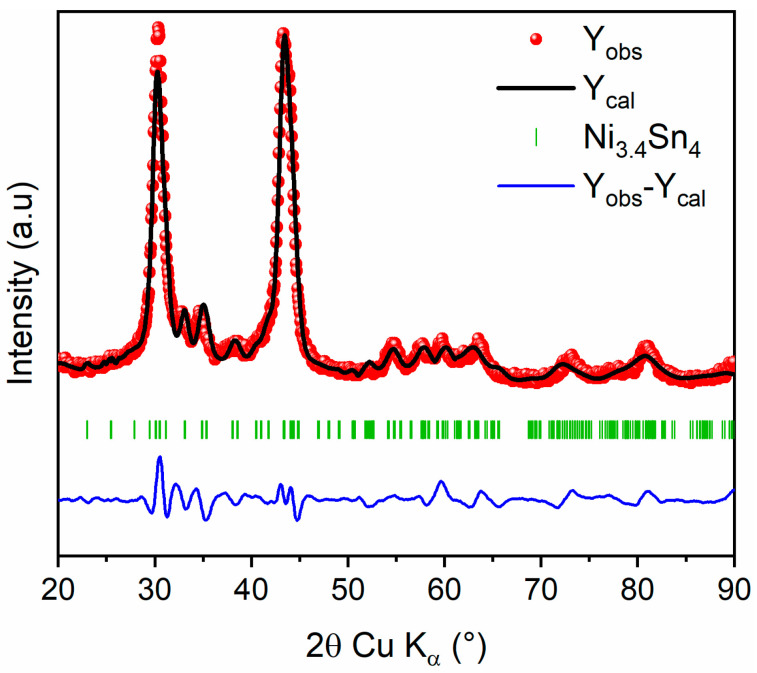
Rietveld refinement of XRD pattern of 20 h milled Ni_3.4_Sn_4_: Observed (red dots), calculated (black line), and difference (blue line) curves are shown. The vertical marks show the Bragg positions for Ni_3.4_Sn_4_ phase.

Afterward, the evolution of XRD patterns of Ni_3.4_Sn_4_ with milling time is depicted in Figure S2 and Table S2. The Ni_3.4_Sn_4_ phase is preserved during milling with gradual broadening of all diffraction peaks. The refined cell parameters and crystallite sizes are gathered in Table S1. Based on previous findings, the 20 h milled Ni_3.4_Sn_4_ sample was selected for further investigation owing to its superior electrochemical performance [22]. The refined XRD pattern for 20 h milled Ni_3.4_Sn_4_ and the corresponding refined structural parameters are presented in Figure 4 and Table 3, respectively. The unit cell parameters align well with those previously reported [22]. The slight expansion may be attributed to the plastic deformation induced by ball milling, which also leads to a significant reduction in crystallite size, down to ~7 (1) nm, as inferred from the large peak broadening.

The average chemical composition of 20 h milled Ni_3.4_Sn_4_, as determined by ICP analysis, is gathered in Table 3. A minor Fe contamination from the milling tools, ≤0.09 at.%, was detected. The measured atomic ratio between Ni and Sn concurs fairly well with the targeted values for Ni_3.4_Sn_4_.

**Figure 5:**
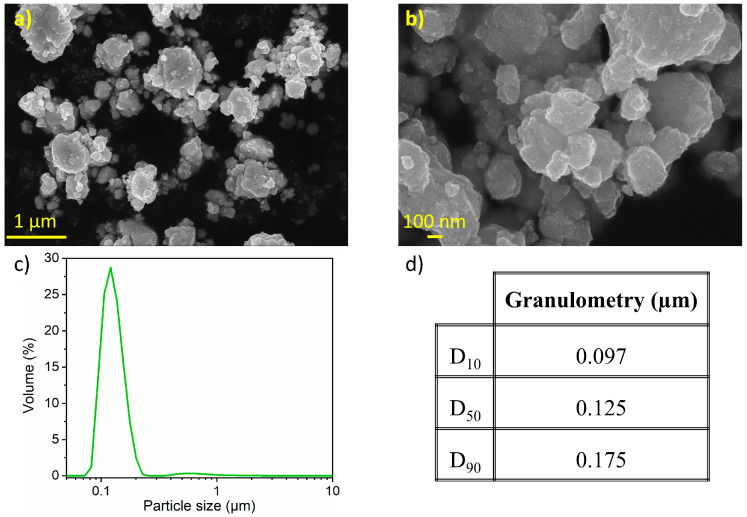
(**a**,**b**) Secondary electron image with in-lens detector with two magnifications; (**c**) particle size distribution curve from LD measurements; and (**d**) particle size percentiles of the 20 h milled Ni_3.4_Sn_4_.

Figure 5 presents the SEM morphology of 20 h milled Ni_3.4_Sn_4_. The sample consists of secondary particles with an average size of ~1 μm (Figure 5a). At higher magnification, the size of primary particles can be estimated to range from 100 to 200 nm (Figure 5b), matching the particle size distribution in Figure 5c. The D_10_, D_50_, and D_90_ values indicate a main particle size of 0.15 µm (Figure 5d). It is worth noting that the LD analysis only captures the primary particle sizes, with no indication of larger agglomerates likely due to their disruption by ultrasonic agitation or their weight preventing suspension during analysis.

### 3.2. Li_6_PS_5_Cl Solid Electrolyte

#### 3.2.1. Structural and Morphological Characterization

**Figure 6:**
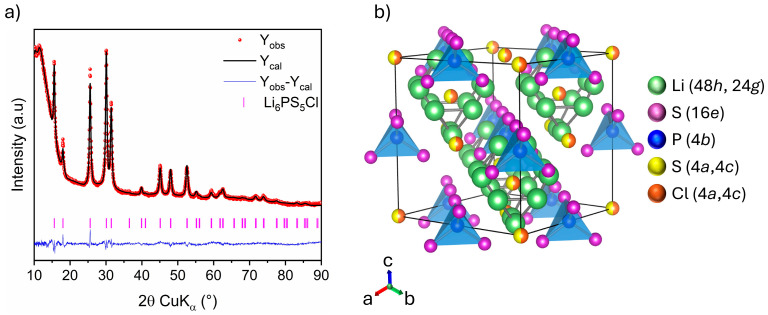
(**a**) Rietveld refinement of XRD pattern: Observed (red dots), calculated (black line), and difference (blue line) curves are shown. The vertical marks show the Bragg positions for Li_6_PS_5_Cl phase, (**b**) Crystal structure schema of Li_6_PS_5_Cl.

Rietveld analysis of the XRD pattern of Li_6_PS_5_Cl chlorine argyrodite is displayed in Figure 6a, with the corresponding crystallographic data gathered in Table 4. The halo patterns at low angles result from the dome-like X-ray transparent cap used to prevent the sample from air exposure. Rietveld refinement indicates that Li_6_PS_5_Cl crystallizes in a cubic system with the $F\bar{4}3m$ space group. The lattice parameter and average crystallite size are determined to be 9.8495 (5) Å and 15 (1) nm, respectively, consistent with previously reported values [29,30]. The crystal structure of Li_6_PS_5_Cl, illustrated in Figure 6b, reveals a cubic-close-packed lattice. The backbone consists of PS_4_^−^ polyhedra centered at 4b sites and formed by S^2−^ at 16*e* sites. The remaining S^2−^ and Cl^−^ occupy the 4*a* and 4*c* sites, of which approximately 50% of the surrounding 48*h* sites are occupied by lithium-ions. The lithium distribution across the 48*h* and 24*g* sites creates a cage-like structure centered at 4*c* sites. In this context, molecular dynamic simulations revealed that the occupancy ratio of halide anions on 4*a* and 4*c* sites (disorder) significantly influences the frequency of lithium-ion mobility in the argyrodite lattice [31,32,33,34]. This, in turn, affects the argyrodite’s overall ionic conductivity. Unfortunately, S^2−^/Cl^−^ occupancies were not refined for disorder in our case since Cl^−^ and S^2−^ are indistinguishable via XRD due to their similar scattering factors [33].

**Figure 7:**
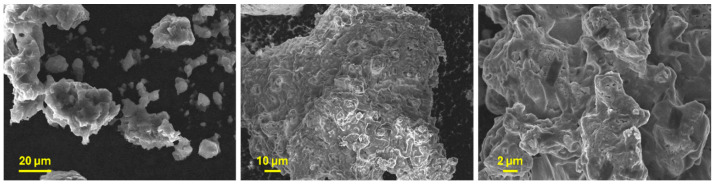
SEM micrographs at different magnifications showing the morphology of Li_6_PS_5_Cl powder.

The SEM micrographs in Figure 7 show that Li_6_PS_5_Cl, obtained through mechanical milling, is composed of coal-grain-like particles displaying irregular shapes and sizes, with a propensity to agglomerate. This latter underscores the ductile nature of the argyrodite.

#### 3.2.2. Electrochemical Characterization

**Figure 8:**
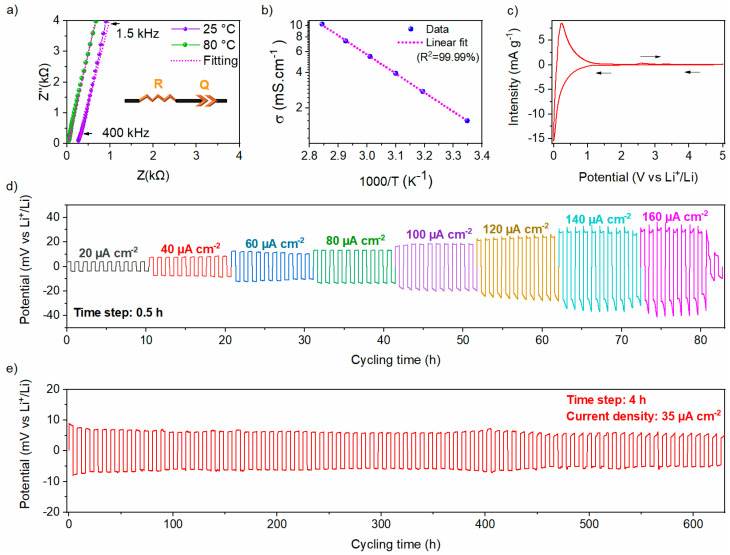
(**a**) Nyquist plots at 25 °C and 80 °C with the corresponding equivalent circuit, (**b**) Arrhenius plot from EIS measurements, (**c**) Cyclic voltammetry curve with a scan rate of 10 µV s^−1^ of Li_6_PS_5_Cl. Voltage responses of the Li|Li_6_PS_5_Cl|Li symmetric cell under repeated polarization with (**d**) rate capability from 20 to 160 μA cm^−2^ with 0.5 h per step, and (**e**) under a constant current density of 35 μA cm^−2^ with 4h per step.

Ionic conductivity is one of the uppermost properties of a solid electrolyte. Nyquist plots of EIS for the Li_6_PS_5_Cl electrolyte at 25 and 80 °C are shown in Figure 8a. The impedance data are mostly composed of a steep linear spike attributed to a blocking behavior between the Li_6_PS_5_Cl conductor and the carbon electrodes. The impedance data were fit with an R-Q series equivalent circuit model, illustrated in the inset of Figure 8a, where R stands for the ionic resistance of the solid electrolyte, and Q represents the constant phase element (CPE) used to model the non-ideal capacitor. The ionic conductivity of the Li_6_PS_5_Cl solid electrolyte increases from 1.5 mS cm^−1^ at 25 °C to 10.2 mS cm^−1^ at 80 °C, consistent with reported values for the chlorine argyrodite [29,30]. The shift of the Nyquist plots toward higher frequencies with increasing temperature, as more clearly displayed in Figure S3a, is attributed to Arrhenius-type thermally activated ionic transport phenomena. For clarity, the high-frequency regions at 25 °C and 80 °C are enlarged in Figure S3b. The thermal dependence of the ionic conductivity, as shown in Figure 8b, gives an activation energy of 0.32 (1) eV, aligning with previously reported values [30,34,35]. The electronic conductivity at RT of the Li_6_PS_5_Cl solid electrolyte is found to be 3.2 (5) × 10^−5^ mS cm^−1^, calculated using the DC chronoamperometry method (Figure S3c).

To study the electrochemical window stability of Li_6_PS_5_Cl chlorine argyrodite, a CV measurement was performed as displayed in Figure 8c. A reversible redox activity toward lithium was observed within the electrochemical window between 0 and 5 V vs. Li^+^/Li. A pronounced reduction peak is observed within the 0–0.5 V vs. Li^+^/Li potential range, indicating significant degradation of the chlorine argyrodite, which appears to be partially reversible, as evidenced by the prominent oxidation peak observed at 0.23 V vs. Li^+^/Li. Afterward, a small oxidation peak appears at 2.6 V vs. Li^+^/Li, with a corresponding reduction peak at 1.5 V vs. Li^+^/Li, which is due to a limited redox decomposition of the chlorine argyrodite electrolyte [36]. Beyond these observations, no other redox peaks were detected, indicating that the electrolyte can be effectively coupled with active materials operating at various potentials.

The electrochemical stability of Li_6_PS_5_Cl toward lithium metal was evaluated using a Li|Li_6_PS_5_Cl|Li symmetric cell at RT and measured in terms of critical current density (CCD) and rate capability of lithium-ions plating/stripping performance. As displayed in Figure 8d, the symmetric cell was evaluated by step-ascending current densities with a step gap of 20 µA cm^−2^. The polarization voltage increases with the applied current density, according to Ohm’s law. From 20 to 140 µA cm^−2^, the potential increases during the plating/stripping step. The total resistance was determined to be 661 ± 55 Ω, which exceeds the intrinsic resistance of the bulk Li_6_PS_5_Cl estimated at 255 ± 5 Ω. This discrepancy can be attributed to the formation of a resistive interfacial layer upon contact with lithium metal, arising from the electrochemical instability of argyrodite at extremely low potentials. At a current density of 160 µA cm^−2^, an internal short circuit of the cell was recorded, indicated by a sharp decrease of the overpotential. Thus, the CCD is determined to be 140 µA cm^−2^ at RT.

Afterward, the long-term stability of lithium plating and stripping was evaluated by cycling a Li|Li_6_PS_5_Cl|Li symmetric cell at a constant current density of 35 µA cm^−2^ at RT. This current density was selected based on the conditions under which the solid-state half-cell was cycled in this study (~5 mA g^−1^). The voltage profile and magnified voltage profiles are shown in Figure 8e and Figure S4, respectively. The symmetric cell demonstrated a flat axisymmetric polarization curve, suggesting highly stable and reversible lithium plating/stripping behavior at the Li/Li_6_PS_5_Cl interface over 600 hours. It is worth mentioning that Li_6_PS_5_Cl exhibits a relatively stable polarization voltage of 8.2 (±0.5) mV over 600 h of cycling, indicating good stability at the interface between Li_6_PS_5_Cl and lithium under the tested conditions.

### 3.3. Electrochemical Characterization of Sn-Based All-Solid-State Half-Cell

Assembling an ASSLiB using solid electrolyte presents significant challenges, notably regarding chemical compatibility within the composite electrode. Thus, the compatibility of the intimate mixture of each anode active material and Li_6_PS_5_Cl, aged for two weeks at RT, was investigated by XRD analysis. Rietveld refinement of the XRD patterns for both composites is shown in Figure S5 with the corresponding refined structural parameters gathered in Table S3. The calculated patterns correspond to the sum of the individual components’ patterns, with no crystalline impurity phases observed. This suggests that no massive reaction occurs between the active material and solid electrolyte after mixing and aging at RT. These preliminary tests indicate that Li_6_PS_5_Cl is likely chemically compatible as a solid electrolyte with SnHPO_3_ and Ni_3.4_Sn_4_ electrode active materials.

Solid-state half-cells were cycled in galvanostatic mode at a constant current of 5 mA g^−1^ within the potential ranges of [0.1–2] and [0.2–2] V vs. Li^+^/Li for SnHPO_3_ and Ni_3.4_Sn_4_ respectively. It is crucial to point out that the lower voltage cutoff was set above 0 V vs. Li^+^/Li to mitigate the degradation of the argyrodite electrolyte. Consequently, an incomplete lithiation of SnHPO_3_ and Ni_3.4_Sn_4_ occurs, resulting in a predicted initial capacity reduction of approximately 10% and 40% of the solid-state half-cells compared with their LP30 liquid counterparts.

**Figure 9:**
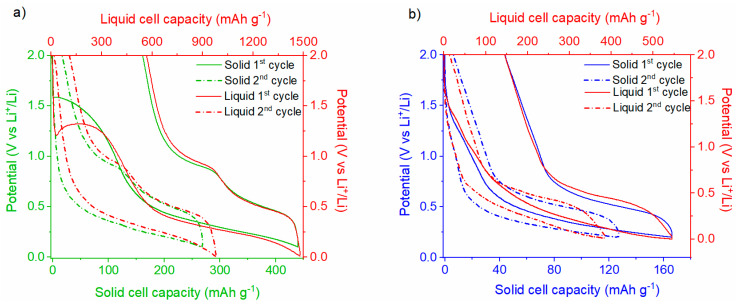
First and second galvanostatic discharge/charge profiles of (**a**) Li|Li_6_PS_5_Cl|SnHPO_3_ and (**b**) Li|Li_6_PS_5_Cl|Ni_3.4_Sn_4_ half-cells at 5 mA g^−1^ in comparison with their corresponding liquid half-cells at 108 mA g^−1^.

The voltage profiles for the 1st and 2nd cycles of the Li|Li_6_PS_5_Cl|SnHPO_3_ solid half-cell, compared with those of the Li|LP30| SnHPO_3_ liquid half-cell, are presented in Figure 9a. The potential profiles for the solid half-cell exhibit an analogous trend to that observed in the liquid half-cell. Albeit the variations in the extent of the lithiation reaction between both cells, they exhibit a similar reaction mechanism as better displayed in Figure S6a. Considering the solid-state half-cell, an irreversible pseudo-plateau emerges at ~1.5 V vs. Li^+^/Li during the first lithiation, which is notably higher than that observed in the liquid half-cell (~1.3 V vs. Li^+^/Li). This plateau, involving the exchange of 0.7 Li^+^ per SnHPO_3_ (100 mAh g^−1^), is attributed to the irreversible reduction of a fraction of Sn^(II)^ in SnHPO_3_ to metallic tin Sn^(0)^, as reported in previous studies [23]. Notably, this plateau is not related to the solid electrolyte interphase (SEI) formation since the cyclic voltammogram of chloride argyrodite demonstrates its electrochemical stability at this potential. Following the first lithiation, an extended plateau down to 0.1 V vs. Li^+^/Li corresponds to the alloying process of the metallic tin, resulting in the formation of Li*_x_*Sn alloys. A total of 3.2 Li^+^ per SnHPO_3_ is exchanged during this process, with the initial lithiation capacity reaching 440 mAh g^−1^, much lower than the 1480 mAh g^−1^ achieved in the liquid half-cell. During the subsequent delithiation, two distinct plateaus at 0.48 and 0.90 V vs. Li^+^/Li are observed, attributed to the dealloying of Li*_x_*Sn alloys [23]. The initial reversible capacity for the solid half-cell is 280 mAh g^−1^, corresponding to 2.1 Li^+^ per SnHPO_3_. The irreversible capacity fraction, approximately 40%, is comparable to that of the liquid cell, as it is mainly ascribed to the irreversible reduction in Sn^(II)^ to Sn^(0)^ and SEI formation at low voltages.

The voltage profiles for the 1st and 2nd cycles of the Li|Li_6_PS_5_Cl|Ni_3.4_Sn_4_ solid-state half-cell compared with the Li|LP30|Ni_3.4_Sn_4_ liquid half-cell are shown in Figure 9b. The initial galvanostatic cycles for both the liquid and solid half-cells display no significant differences, indicating similar electrochemical behavior despite the lithiation extent, as displayed in Figure S6b. Starting the lithiation of the solid half-cell, the voltage quickly drops to 1.2 V vs. Li^+^/Li, followed by an initial potential step that is associated with the formation of the SEI interphase, as reported elsewhere [22]. Subsequently, a broad plateau at [0.2–0.6] V vs. Li^+^/Li potential range is observed, corresponding to the reversible formation of Li*_x_*Sn alloys. Upon the subsequent delithiation, a prominent plateau at 0.5 V vs. Li^+^/Li is discernible and attributed to the deformation of Li*_x_*Sn alloys. From the 2nd cycle onward, the potential profiles show no further signs of SEI formation and, similarly to the liquid half-cell, remain smooth. The 1st cycle shows a reversible capacity of 120 mAh g^−1^, which is lower than the 400 mAh g^−1^ observed in the liquid half-cell.

**Figure 10:**
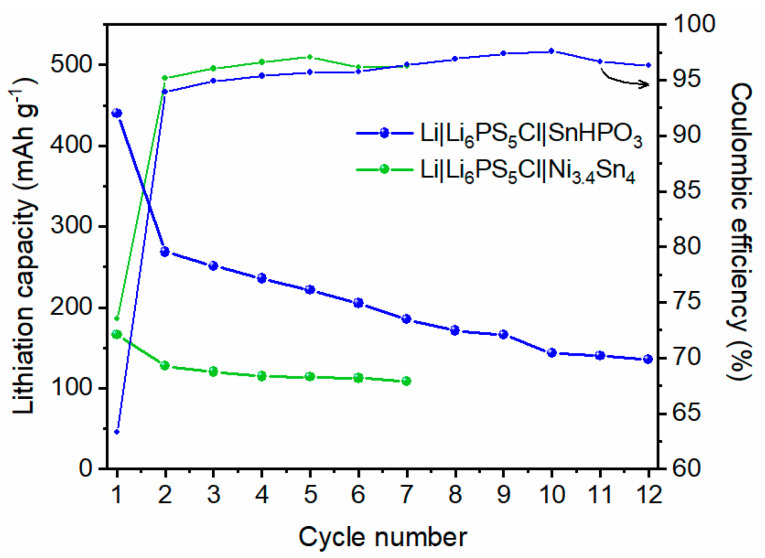
Evolution of the specific lithiation capacity and coulombic efficiency of Li|Li_6_PS_5_Cl|SnHPO_3_ and Li|Li_6_PS_5_Cl|Ni_3.4_Sn_4_ solid-state half-cells during galvanostatic cycling at 5 mA g^−1^.

The evolution of lithiation capacity and coulombic efficiency over multiple cycles for the two solid-state half cells is depicted in Figure 10. The evolution of galvanostatic profiles is shown in Figure S7. For SnHPO_3_ material, a significant capacity decay is observed during the 1st cycle, followed by a gradual decline over the subsequent cycles. In contrast, the Ni_3.4_Sn_4_ material begins with a lower lithiation capacity of 170 mAh g^−1^; nonetheless, it maintains a more stable capacity on cycling, showing better capacity retention. The coulombic efficiencies improve significantly by the 2nd cycle, stabilizing above 95% for SnHPO_3_ and 97% for Ni_3.4_Sn_4_.

## 4. Discussion

In this study, the conversion alloying-based materials SnHPO_3_ and Ni_3.4_Sn_4_ were tested for the first time as anode materials for ASSLiBs using argyrodite Li_6_PS_5_Cl as a solid electrolyte. Ball milling yielded nanostructured SnHPO_3_ (24(1) nm)and Ni_3.4_Sn_4_ (7(1) nm) after 1 h and 20 h of milling, respectively. The obtained nanometric primary particles are crucial for ASSLiBs, as they shorten lithium-ion diffusion paths within the active material, minimizing internal resistance.

The morphological analysis of the chlorine argyrodite revealed a ductile nature, facilitating its mechanical interlocking with the active material. Additionally, the solid electrolyte exhibits high ionic conductivity (up to 1.5 mS cm^–1^) with negligible electronic conductivity. Interestingly, although neutron diffraction data to quantitatively assess the structural disorder are unavailable, this exceptional ionic conductivity is ascribed to the presence of antisite disorder between the 4*a* and 4*c* crystallographic sites, which facilitates enhanced lithium-ion mobility by generating additional diffusion pathways. Moreover, these properties were achieved through a simple 10 h ball milling process, offering performance comparable to methods involving extended milling (>24 h) and/or high-temperature sintering (>550 °C). This synthesis approach is, therefore, both efficient and cost-effective, requiring less energy and equipment compared with the reported methods [1,30,37,38]. Furthermore, the symmetric cell exhibited a CCD of 140 μA cm^−2^, representing a limiting factor for the application of high C-rates in subsequent solid-state half-cells. In this regard, it is important to emphasize that the symmetric cell was cycled without external pressure, as was the case for all manipulations in this study. This condition may lead to local current inhomogeneities, promote dendritic lithium growth, and ultimately accelerate short-circuiting, thereby contributing to the relatively moderate CCD observed [1,2,5,31,35].

The electrochemical performances of SnHPO_3_ and Ni_3.4_Sn_4_ in solid-state half-cells were compared with those in liquid half-cells. The potential profiles for the solid-state half-cells exhibit a trend similar to that of the liquid half-cells, suggesting that both systems undergo the same electrochemical mechanisms. This is an encouraging result, as it indicates that switching from liquid to solid electrolytes does not alter the fundamental electrochemical behavior of the active materials. Moreover, more broadly, the electrochemical profiles are in excellent agreement with those reported for other Sn-based negative electrodes in ASSLiBs [8,15,21]. However, the first lithiation capacities were 440 mAh g^−1^ for SnHPO_3_ and 170 mAh g^−1^ for Ni_3.4_Sn_4_, both significantly lower than the capacities achieved in liquid counterparts. It is important to remember that the nanostructured SnHPO_3_ demonstrated an initial capacity of 1480 mAh g^−1^ at 108 mA g^−1^ in a liquid half-cell, attributed not only to the conversion-alloying mechanism reported in Equations (1) and (2), but also to a surface-related supercapacitive contribution and a possible Sn overoxidation upon delithiation [23]. The conversion process is, however, limited in the solid half-cell (0.7 Li^+^ per SnHPO_3_) as compared with the liquid half-cell, which involves the exchange of 2.7 Li^+^ per SnHPO_3_. In the liquid half-cell, a complete conversion of Sn^(II)^ to Sn^(0)^ occurs, accompanied by the formation of the SEI layer. This process results in the insertion of ~2.7 Li^+^ during the conversion plateau at potentials exceeding 1.1 V vs. Li^+^/Li. In contrast, the conversion process is significantly limited in the solid-state half-cell, with only 0.7 Li^+^ exchanged per SnHPO_3_ unit and no SEI formation arising from argyrodite degradation. This observation suggests that only a fraction of SnHPO_3_ (~35%) undergoes conversion to metallic tin, as described by Equation (1). Consequently, the total reversible capacity in the solid-state half-cell is reduced by a factor of three, decreasing from 1480 to 440 mAh g^−1^. The limited ductility of chlorine argyrodite Li_6_PS_5_Cl is seemingly insufficient to accommodate the volume changes associated with the conversion process, thereby resulting in partial conversion.

(1) $${{\mathrm{Sn}}^{\left(\mathrm{II}\right)}\mathrm{HPO}}_{3}+{\;2\;\mathrm{Li}}^{+}+{2\;e}^{-}⟶{\mathrm{Sn}}^{\left(0\right)}+{\;\mathrm{Li}}_{2}{\mathrm{HPO}}_{3}$$

(2) $$\mathrm{Sn}+{\;x\;\mathrm{Li}}^{+}+{x\;e}^{-}⇌{\;\mathrm{Li}}_{x}\mathrm{Sn}\;(0\;<\;x\;<\;4.4)$$

As for the 20 h-milled Ni_3.4_Sn_4_, it delivered an initial lithiation capacity of 546 mAh g^−1^ at 0.2 C in a liquid half-cell compared with 166 mAh g^−1^ in the solid-state counterpart. This capacity results from a two-step lithiation process enabled by the conversion-alloying mechanism gathered in Equation (3) [22].

(3) $${\mathrm{Ni}}_{3.4}{\mathrm{Sn}}_{4}+{x\;\mathrm{Li}}^{+}+{x\;e}^{-}⟶{\mathrm{Li}}_{x}\mathrm{Sn}+3.4\;\mathrm{Ni}$$

A possible reason for the reduced initial lithiation capacities obtained in the solid-state half-cells may be the low cutoff potential set well above 0 V vs. Li^+^/Li, a methodical choice made to mitigate the degradation of the Li_6_PS_5_Cl argyrodite electrolyte at low potential. This precaution inevitably limits the lithiation extent of SnHPO_3_ and Ni_3.4_Sn_4_, resulting in the anticipated capacity reductions of approximately 40% and 10%, respectively, based on liquid half-cell galvanostatic cycling. However, the solid-state half-cells reveal a more pronounced reduction of approximately 70% for both materials. This finding suggests that the lower cutoff potential cannot be considered the sole factor limiting capacity in the solid-state half-cells. Specifically, the performance gap points to the influence of structural challenges within the composite electrode architecture on the accessible charge storage.

According to this approach, the low initial capacities observed in solid-state half-cells may result from the formation of isolated or disconnected regions of active material within the composite electrode. These disjointed regions hinder the efficient transport of lithium-ions and electrons throughout the composite electrode. Within an ideal composite electrode architecture, interconnected pathways are induced by lithium-ions and electron-conducting phases, leading to efficient charge transfer. Disconnected regions, on the other hand, lead to incomplete conductive pathways, where lithium-ions may be trapped, and electrons may not reach all active sites, resulting in poor utilization of the active material. As part of this investigation, the utilization of both active materials was assessed through SEM/EDX morphological characterization of each composite anode prior to cycling. The SEM images and phase mappings are presented in (Figures S8 and S9). For both electrodes, SEM analysis reveals composite particles consisting of distinct phase domains: the light-toned regions are attributed to the anode active material (SnHPO_3_ or Ni_3.4_Sn_4_) embedded within a dark-grey matrix corresponding to the argyrodite and VGCF matrix. The ductile nature of the argyrodite is evident; however, the anode active material appears as agglomerates. This agglomeration hinders electrochemical percolation within the composite anode, prolonging diffusion paths to the bulk of large particle agglomerates and resulting in portions of the active material being isolated from the argyrodite. Consequently, these isolated regions do not contribute to the overall capacity, leading to reduced electrochemical performance.

The initial coulombic efficiency (irreversible capacity) observed in the solid half-cells is of the same order of magnitude as that of the liquid half-cells. One potential contributor is the degradation of the active materials, which may arise from the substantial volume expansion of Sn during lithiation [20]. This expansion can lead to pulverization, cracking, and stress generation within the active material, which compromises the structural integrity of the electrode, resulting in irreversible disconnection between the electrode and the solid electrolyte. Another plausible factor is the formation of an SEI interphase at the interface between the Li_6_PS_5_Cl solid electrolyte and the electrode at low potential [5,39]. The SEI layer is thus composed of the decomposition products of the solid electrolyte consuming lithium and contributing as well to the irreversible capacity.

Upon cycling, both solid-state half-cells exhibit lower reversible capacity compared with their corresponding liquid half-cells. Nonetheless, despite the reduced performance, the reversible capacity of the Li|Li_6_PS_5_Cl|Ni_3.4_Sn_4_ solid half-cell stabilizes at 110 mAh g^−1^ with a coulombic efficiency exceeding 97%. In contrast, a more pronounced degradation is observed for the Li|Li_6_PS_5_Cl| SnHPO_3_ solid half-cell, pointing to specific challenges that may arise from the intrinsic properties of SnHPO_3_. In fact, the more substantial volume changes experienced by SnHPO_3_ during the lithiation/delithiation process are according to its higher expected capacity. This structural instability can lead to increased mechanical stress, which may loosen the contact between the active material and the solid electrolyte. Moreover, the reduced performance may come from a substantial continuous degradation of Li_6_PS_5_Cl triggered by the presence of a higher amount of carbon (10 wt.%) in the composite electrode. This thoughtful choice made for enhancing SnHPO_3′_s electronic conductivity [23] may have accelerated the decomposition of Li_6_PS_5_Cl, as the extent of solid electrolyte degradation correlates with carbon content [40,41]. Furthermore, it is important to note the studied solid-state cells were cycled without external pressure. In the absence of sufficient pressure, the repeated expansion and contraction of the active materials can loosen the contact between the active material and the solid electrolyte, impeding lithium-ion transport and leading to electrode fragmentation. These findings could be further clarified through in-depth morphological characterization of the interface between the argyrodite and the working electrode. Unfortunately, this was not possible in the current study, as the bilayer pellet architecture prevents post-mortem analysis without compromising the integrity of the system. Future studies focusing on the evolution of solid-solid interfaces represent a promising direction for further investigation.

## 5. Conclusions

This study demonstrates the first investigation of conversion/alloying materials SnHPO_3_ and Ni_3.4_Sn_4_ as potential anode materials for ASSLiBs employing Li_6_PS_5_Cl as the solid electrolyte. The ball milling produced nanostructured materials (24(1) nm for SnHPO_3_ and 7(1) nm for Ni_3.4_Sn_4_), enhancing lithium-ion diffusion and reducing mechanical stress in the composite electrode. Despite the similarity in electrochemical reaction mechanisms to those observed in liquid half-cells, the extent of lithiation in solid-state half-cells remains limited, yielding initial capacities of 440 mAh g^−1^ (SnHPO_3_) and 170 mAh g^−1^ (Ni_3.4_Sn_4_). The reaction mechanism is the same as the liquid cells can be operated upon without any external pressure; the extent of the lithiation reaction is lower for solid cells with initial capacities of 440 mAh g^−1^ (SnHPO_3_) and 170 mAh g^−1^ (Ni_3.4_Sn_4_). This capacity reduction goes beyond the limited low-potential cutoff, underlining additional limitations such as the agglomeration of the active material within the electrode, leading to isolated regions that impede lithium-ion transport. Mechanical degradation, particularly with SnHPO_3_, further exacerbates these drawbacks. With its higher lithiation capacity, SnHPO_3_ undergoes higher volume changes during cycling, generating substantial stress.

These results, obtained without the application of external pressure, highlight the need to optimize pressure conditions to prevent electrode fragmentation and ensure optimal interfacial contact between the active material and the solid electrolyte. Future studies should focus on refining electrode architecture to enhance the electrochemical performance and long-term stability of Sn-based anodes in ASSLiBs. Additionally, the lower cutoff potential should be carefully adjusted to allow sufficient lithiation while maintaining the electrochemical stability of the argyrodite at low potential. Further exploration of alternative composite electrode preparation methods, such as high-energy ball milling with controlled parameters or chemical routes, could refine the particle size distribution between the active material and the argyrodite solid electrolyte, thereby improving electrochemical kinetics. These research directions provide a framework for future investigations, fostering the advancement of Sn-based anodes for next-generation ASSLiBs.

## Figures and Tables

**Table 1:** Refined structural parameters by Rietveld method of nanostructured SnHPO_3_. Rietveld agreement factors (R_Bragg_, R_p_, R_wp_, and χ^2^) are provided. Standard deviations referred to the last digit are given in parentheses.

Unit Cell Parameters				Density (g cm^−3^)	Crystallite Size (nm)
*a* (Å)	*b* (Å)	*c* (Å)	*β* (°)		
7.0730 (2)	12.3153 (4)	4.6802 (2)	120.894 (2)	3.7	24 (2)
R_Bragg_ = 3.2	R_p_ = 12.6		R_wp_ = 9.5		χ^2^ = 2.3

**Table 2:** Chemical composition determined by ICP analysis of non-milled and 20 h milled Ni_3.4_Sn_4_.

Sample	Metal-Content			ICP Composition
	at.% Ni	at.% Sn	at.% Fe	
Non-milled Ni_3.4_Sn_4_	46.35	53.61	0.04	Ni_3.5(1)_Sn_4_
20 h milled Ni_3.4_Sn_4_	46.62	53.29	0.09	

**Table 3:** Refined structural parameters of 20 h milled Ni_3.4_Sn_4_. Rietveld agreement factors (R_Bragg_, R_p_, R_wp_, and χ^2^) are provided. Standard deviations referred to the last digit are given in parentheses.

Unit Cell Parameters				Density (g cm^−3^)	Crystallite Size (nm)
*a* (Å)	*b* (Å)	*c* (Å)	*β* (°)		
12.5053 (7)	4.0872 (2)	5.2342 (3)	103.917 (9)	8.5	7 (1)
R_Bragg_ = 5.7	R_p_ = 17.5		R_wp_ = 17.7		χ^2^ = 4.6

**Table 4:** Refined crystallographic data of Li_6_PS_5_Cl. Rietveld agreement factors (R_Bragg_, R_p_, R_wp_, and χ^2^) are provided. Standard deviations referred to the last digit are given in parentheses.

	Unit Cell Parameter *a* (Å)		Density (g cm^−3^)	Crystallite Size (nm)	
	
	9.8495 (5)		1.9	15 (1)	
	R_Bragg_ = 2.9	R_p_ = 15.4	R_wp_ = 11.7	χ^2^ = 2.3	
Atom	Wyckoff Site	Atomic Positions			SOF
		*x*	*y*	*z*	
**Li**	48*h*	0.1989	0.1989	0.0025	0.44
**Li**	24*g*	0.0538	0.2500	0.2500	0.12
**S**	16*e*	0.6204 (3)	0.6204 (3)	0.6204 (3)	1
**Cl**	4*c*	14	14	14	1
**S**					
**P**	4*b*	12	12	12	1
**S**	4*a*	0	0	0	1
**Cl**					

## Data Availability

The original contributions presented in this study are included in the article/Supplementary Material. Further inquiries can be directed to the corresponding author.

## Supplementary Materials

The following supporting information can be downloaded at: https://www.mdpi.com/article/10.3390/nano15070512/s1, Figure S1: Rietveld refinement of XRD pattern of non-milled Ni_3+*x*_Sn_4_. Table S1: Refined crystallographic data of non-milled Ni_3+*x*_Sn_4_. Figure S2: Evolution of (a) XRD patterns and (b) crystallite size of Ni_3.4_Sn_4_ with milling time. Table S2: Evolution of refined cell parameters and crystallite sizes of Ni_3.4_Sn_4_ with milling time. Figure S3: (a) Nyquist plots at different temperatures with the corresponding equivalent circuit, (b) Enlarged Nyquist plots at high frequencies at 25 °C and 80 °C, and (c) current evolution under 0.6 V vs. Li^+^/Li polarization of the Li_6_PS_5_Cl chlorine argyrodite. Figure S4: Magnification of the voltage responses of the Li| Li_6_PS_5_Cl |Li symmetric cell under a constant current density of 35 μA cm^−2^ with 4 h per step. Figure S5: Rietveld refinement of XRD patterns of 1:1 in weight of (a) SnHPO_3_: Li_6_PS_5_Cl and (b) Ni_3.4_Sn_4_: Li_6_PS_5_Cl composites. Table S3: Refined structural parameters by Rietveld method of composites (1:1 in weight SnHPO_3_: Li_6_PS_5_Cl and Ni_3.4_Sn_4_:Li_6_PS_5_Cl). Figure S6: Comparison of the 4th galvanostatic profiles in solid and liquid half-cells for (a) SnHPO_3_ and (b) Ni_3.4_Sn_4_ active materials. Figure S7: Galvanostatic discharge/charge profiles of (a) Li|Li_6_PS_5_Cl|SnHPO_3_ and (b) Li|Li_6_PS_5_Cl|Ni_3.4_Sn_4_ solid-state half-cells. Figure S8: (a) Back-scattered electron micrograph and elemental mapping for SnHPO_3_: Li_6_PS_5_Cl:VGCF/40:50:10 wt.% with (b) Sn (L-edge), (c) S (K-edge), and (d) C (K-edge). Figure S9: (a) Back-scattered electron micrograph and elemental mapping for Ni_3.4_Sn_4_: Li_6_PS_5_Cl:VGCF/78:20:2 wt.% with (b) Sn (L-edge), (c) S (K-edge), and (d) C (K-edge). (See Refs. [24,30,35,42]).
