# Supplementary material for: Exploring the Potential of SnHPO3 and Ni3.4Sn4 as Anode Materials in Argyrodite-Based All-Solid-State Lithium-Ion Batteries
Source: Nanomaterials (Basel). 2025 Mar 28;15(7):512. doi: 10.3390/nano15070512 (PMC11990708; doi:10.3390/nano15070512)
Supplement: Supplementary file 1 [file nanomaterials-15-00512-s001.zip › nanomaterials-3534357-supplementary.pdf]

Exploring the potential of $SnHPO_3$ and $Ni_{3.4}Sn_4$ as anode materials in argyrodite-based all-solid-state lithium-ion batteries

Wissal Tout[1,2], Junxian Zhang[2], Mickael Mateos[2], M'hamed Oubla[1], Fouzia Cherkaoui El Moursli[1], Zineb Edfouf[1], Fermin Cuevas[2*]

[1] *MANAPSE, Faculty of Sciences, Mohammed V University in Rabat, Morocco*

[2] *Univ Paris-Est Creteil, CNRS, ICMPE (UMR 7182), 2 rue Henri Dunant, F-94320 Thiais, France*

**Author to whom correspondence should be addressed.*

Supplementary information

The crystal structure of the intermetallic $Ni_{3+x}Sn_4$ compound have been analysed by XRD analysis. The refined XRD pattern and the corresponding crystallographic data are gathered in Figure S1 and

Table S1 respectively. No crystalline impurity is detected. The compound crystallizes in a monoclinic structure with *C*2/*m* space group.

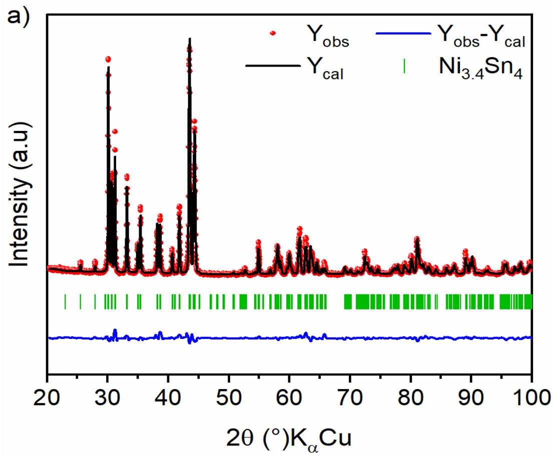

Figure S1 : Rietveld refinement of XRD pattern of non-milled $Ni_{3+x}Sn_4$: Observed (red), calculated (black line) and difference (blue) curves are shown. The vertical marks show the Bragg positions.

Table S1 : Refined crystallographic data of non-milled $Ni_{3+x}Sn_4$. Rietveld agreement factors (R_{Bragg}, R_p, R_{wp} and χ^2) are provided. Standard deviations refereed to the last digit are given in parenthesis.

Unit cell parameters	Atom (Wyckoff site)	Atomic positions			Occupancy
		x	*y*	*z*	

		**Sn (4*i*)**	0.175 (3)	0	0.319 (6)	2
a **(Å)**	12.4110 (1)	**Sn (4*i*)**	0.423 (3)	0	0.197 (6)	2
b **(Å)**	4.0765 (1)	**Ni (4*i*)**	0.783 (5)	0	0.158 (1)	2
c **(Å)**	5.2163 (1)	**Ni (2*c*)**	0	0	$^1/_2$	1
β **(°)**	103.854 (1)	**Ni (2*a*)**	0	0	0	0.47 (2)
R_{Bragg}=10.3	R_p=18.3		R_{wp}=20.1	R_{exp}=6.9		χ^2=8.4

The evolution of the $Ni_{3.4}Sn_4$ XRD patterns with milling time is depicted in Figure S2a, and the refined cell parameters and crystallite sizes are summarized in Table S2. All samples crystallize with monoclinic symmetry in the *C*2/*m* space group. The obtained lattice parameters are in good agreement with the structural data of Furuseth et al [1]. As the milling time increases, a notable broadening of all diffraction peaks is observed, mainly attributed to reduction of crystallite size as displayed in Figure S2b. At the onset of the milling process, the crystallite size decreases rapidly from 180 (5) nm for the non-milled compound to 8 (1) nm after 5 hours of milling. This reduction is primarily due to the severe deformation of powders during initial stages of milling [2]. For extended milling times (> 5 h), the crystallite size stabilizes at 8 (1) nm, indicating a balance between the rate of cold welding and fracturing of powder particles [2].

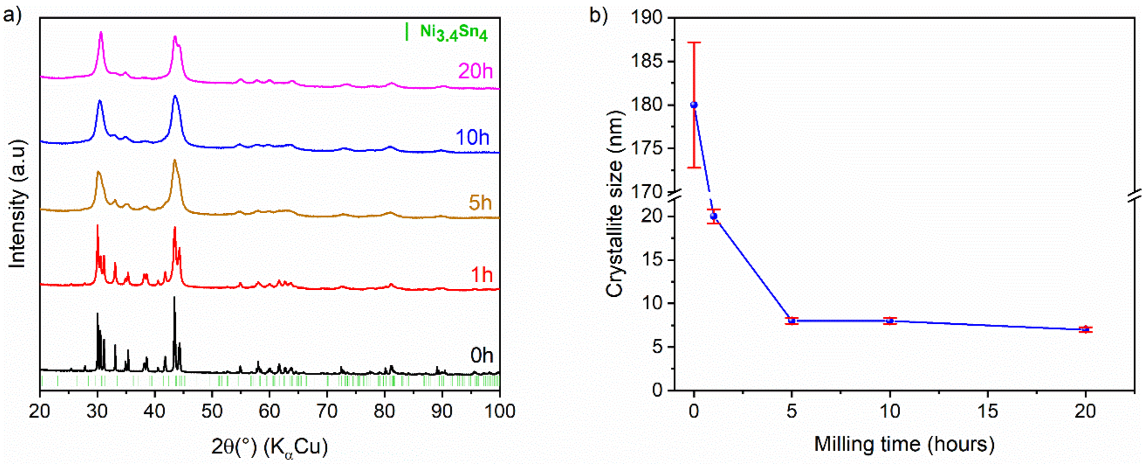

Figure S2 : Evolution of a) XRD patterns and b) average of crystallite size of $Ni_{3.4}Sn_4$ with milling time.

Table S2: Evolution of refined cell parameters and crystallite sizes of $Ni_{3.4}Sn_4$ with milling time. The Rietveld agreement factor R_{bragg} is given. Standard deviations refereed to the last digit are given in parenthesis.

	**0 h**	**1 h**	**5 h**	**10 h**	**20 h**

**Unit cell parameters**	*a* (Å)	12.411 (1)	12.4015 (1)	12.4814 (2)	12.5182 (4)	12.5053 (7)
	b (Å)	4.0765 (1)	4.0774 (1)	4.1073 (1)	4.1053 (1)	4.0872 (2)
	c (Å)	5.2163 (1)	5.2148 (1)	5.2114 (1)	5.2325 (2)	5.2342 (3)
	β (°)	103.854 (1)	103.946 (1)	104.045 (1)	104.05 (2)	103.917 (9)
**Crystallite size (nm)**		180 (7)	20 (1)	8 (1)	8 (1)	7 (1)
$\mathbf{R_{Bragg}}$		10.3	6.7	3.4	5.2	5.7

The impedance spectra of Li_6PS_5Cl solid electrolyte over the measured temperature range are displayed in **Figure S3a**. Curves were fitted using the equivalent circuit shown in the inset of **Figure S3a**. For the sake of clarity, the enlarged Nyquist plots at high frequencies at 25 °C and 80 °C are shown in **Figure S3b**. The linear relationship of log(σ) versus (1/T) follows the Arrhenius law confirming the high purity and thermal stability of Li_6PS_5Cl over the measured temperature range. The activation energy (E_a) for Li^+ conduction was determined from the slope of the linear Arrhenius plot using the simplified equation: $\sigma = A\, e^{-\frac{E_a}{k_B T}}$. where A is the pre-exponential factor T is the absolute temperature, k_B is the Boltzmann constant, and E_a is the activation energy. The determined E_a is 0.32 (1) eV aligning with previously reported values [3,4].

Li_6PS_5Cl electronic conductivity was evaluated from the current observed under a DC polarization at 0.6 V vs Li^+/Li as illustrated in **Figure S3c**. An Li_6PS_5Cl pellet was sandwiched between two stainless steel blocking electrodes in a coin cell following configuration in cell. The resulting electronic conductivity was calculated from Ohm's law to be 3.2 (5) × 10^{-5} mS cm^{-1} indicating that the chlorine argyrodite is an electronic insulator.

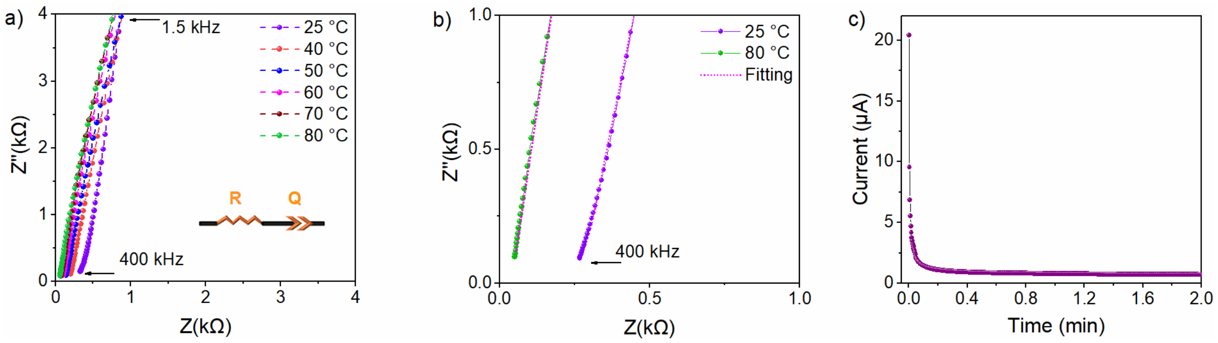

Figure S3 : a) Nyquist plots at different temperatures with the corresponding equivalent circuit, b) Enlarged Nyquist plots at high frequencies at 25 °C and 80 °C and c) current evolution under 0.6 V vs Li^+/Li polarization of the Li_6PS_5Cl chlorine argyrodite.

The voltage responses of the Li|Li_6PS_5Cl|Li symmetric shows stable and symmetric cycling behaviour over time, characteristic of lithium plating/stripping processes. A reproducible electrochemical performance with a low polarization is obtained, indicating good interfacial stability and low resistance at the Li/electrolyte interface. The absence of overpotential increase throughout the extended cycling period implies that Li_6PS_5Cl maintains a stable interface with lithium metal.

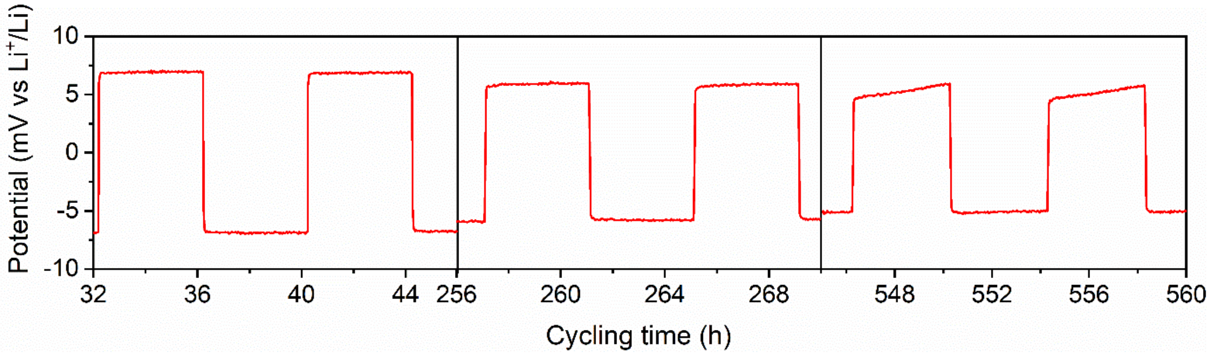

Figure S4 : Magnification of the voltage responses of the Li|Li_6PS5Cl|Li symmetric cell under a constant current density of 35 µA cm^{-2} with 4 h per step.

The chemical reactivity of Li_6PS_5Cl with each anode active material (either $SnHPO_3$ or $Ni_{3.4}Sn_4$) was evaluated by analysing XRD patterns of samples arising from intimate mixing 50 wt.% of Li_6PS_5Cl and 50 wt.% of active material powder ($SnHPO_3$, $Ni_{3.4}Sn_4$). An agate mortar was used to mix the electrolyte with the active material. The mixtures were pelletized to ensure homogeneity allowed to age for two weeks to verify the absence of chemical reactions over time. Afterwards, the pellets were manually ground for the XRD analysis.

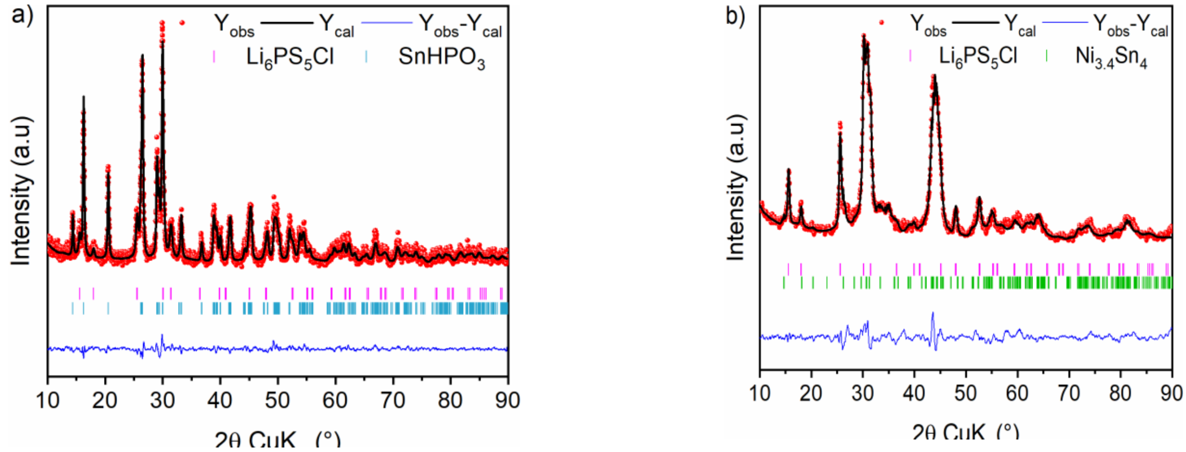

Figure S5 : Rietveld refinement of XRD patterns of 1:1 in weight of (a) $SnHPO_3$:Li_6PS_5Cl and (b) $Ni_{3.4}Sn_4$:Li_6PS_5Cl composites. Observed (red dots). calculated (black line) and difference (blue line below) curves are shown. The blue, green and magenta vertical marks show the Bragg positions for $SnHPO_3$. $Ni_{3.4}Sn_4$ and Li_6PS_5Cl phases respectively.

Table S3: Refined structural parameters by Rietveld method of composites (1:1 in weight $SnHPO_3$:Li_6PS_5Cl and $Ni_{3.4}Sn_4$:Li_6PS_5Cl mixtures). Cell parameters and Rietveld agreement factors (R_{Bragg} and χ^2) are given. Standard deviations refereed to the last digit are given in parenthesis.

	**$SnHPO_3$: Li_6PS_5Cl**		**$Ni_{3.4}Sn_4$: Li_6PS_5Cl**	
	**$SnHPO_3$**	**Li_6PS_5Cl**	**$Ni_{3.4}Sn_4$**	**Li_6PS_5Cl**
**Lattice parameters**	*a* = 7.0816 (5) Å<br>*b*= 12.3129 (9) Å<br>*c*= 4.6855 (3) Å<br>*β*= 120.905 (4) °	*a*= 9.8561 (9) Å	*a*= 12.4382 (7) Å<br>*b*= 4.0740 (8) Å<br>*c*= 5.0373 (5) Å<br>*β*= 104.428 (9) °	a= 9.8501 (4) Å
**Weight fraction (%)**	56 (1)	44 (2)	48 (4)	52 (3)
**R_{Bragg}**	7.6	5.9	7.4	7.3
**χ^2**	1.2		1.5	

The voltage profiles for the 4th cycle of the Li|Li_6PS_5Cl|$SnHPO_3$ and Li|Li_6PS_5Cl|$Ni_{3.4}Sn_4$ solid-state half-cells compared to their liquid counterparts are displayed in Figure S6 a and b respectively. For both materials, the galvanostatic profile displays no significant differences between the solid and liquid half-cells, indicating similar electrochemical behaviour despite the difference in the lithiation extent.

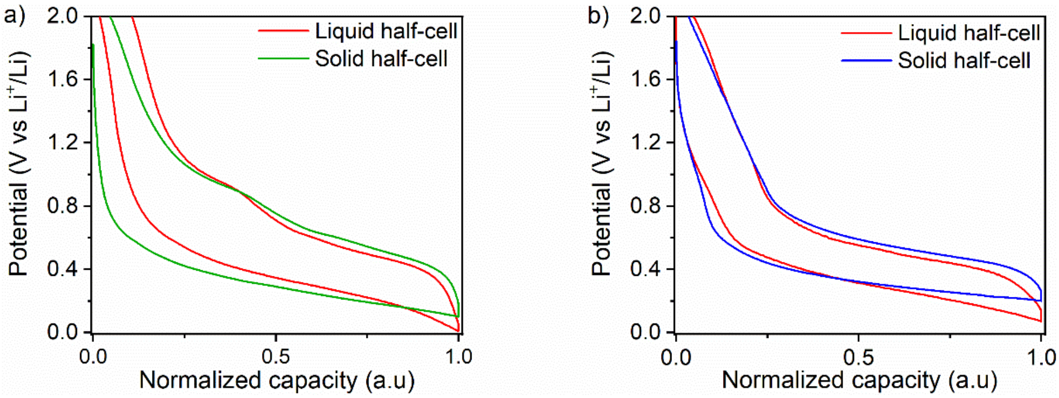

Figure S6 : Comparison of the 4th galvanostatic profiles in solid and liquid half-cells for a) $SnHPO_3$ and b) $Ni_{3.4}Sn_4$ active materials.

Solid-state half-cells were cycled in galvanostatic mode at a constant current of 5 mA g^{-1} within the potential ranges of [0.1-2] and [0.2-2] V vs Li^+/Li for $SnHPO_3$ and $Ni_{3.4}Sn_4$ respectively. The voltage profiles of the Li|Li_6PS_5Cl|$SnHPO_3$ and Li|Li_6PS_5Cl|$Ni_{3.4}Sn_4$ solid-state half-cells are displayed in Figure S7a and b respectively.

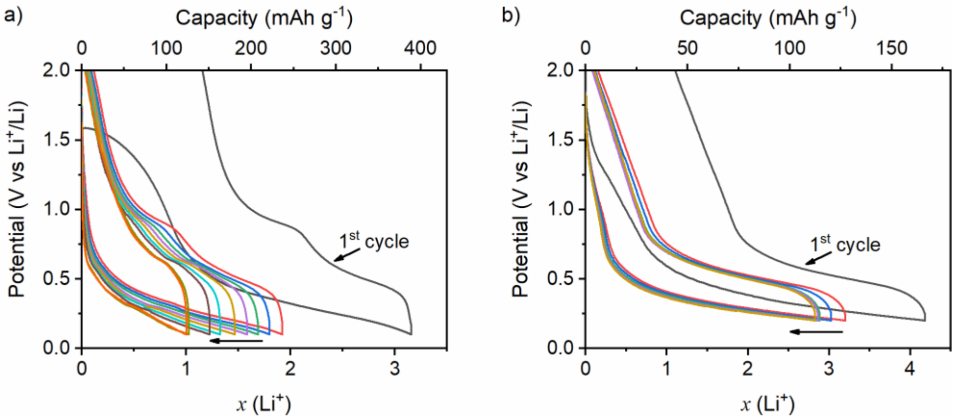

Figure S7 : Galvanostatic discharge/charge profiles of a) Li|Li_6PS_5Cl|$SnHPO_3$ and b) Li|Li_6PS_5Cl|$Ni_{3.4}Sn_4$ solid-state half-cells.

In order to investigate the compatibility of the chlorine argyrodite electrolyte with each $SnHPO_3$ and $Ni_{3.4}Sn_4$ intermetallic, a morphological characterization of the composite electrode was observed before cycling. In the EDX mapping, the distribution of the electrolyte in the composite powder can be tracked through the S element map while the distribution of the active material can be seen through the Sn element map. The VGCF distribution can be seen through C mapping, even though it is not clear due to the carbon affixed in the SEM sample holder.

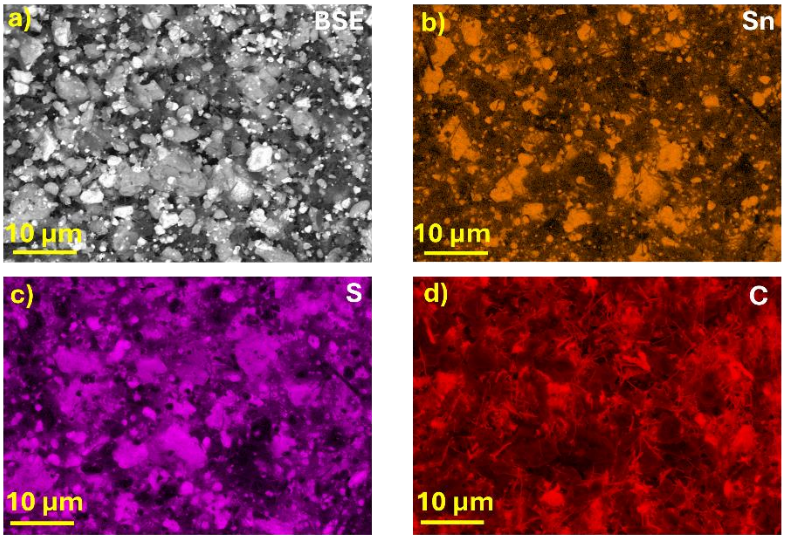

Figure S8: a) Back-scattered electron micrograph and elemental mapping for $SnHPO_3$:Li_6PS_5Cl :VGCF / 40:50:10 $\%_{wt}$ with b) Sn (L-edge). c) S (K-edge) and d) C (K-edge).

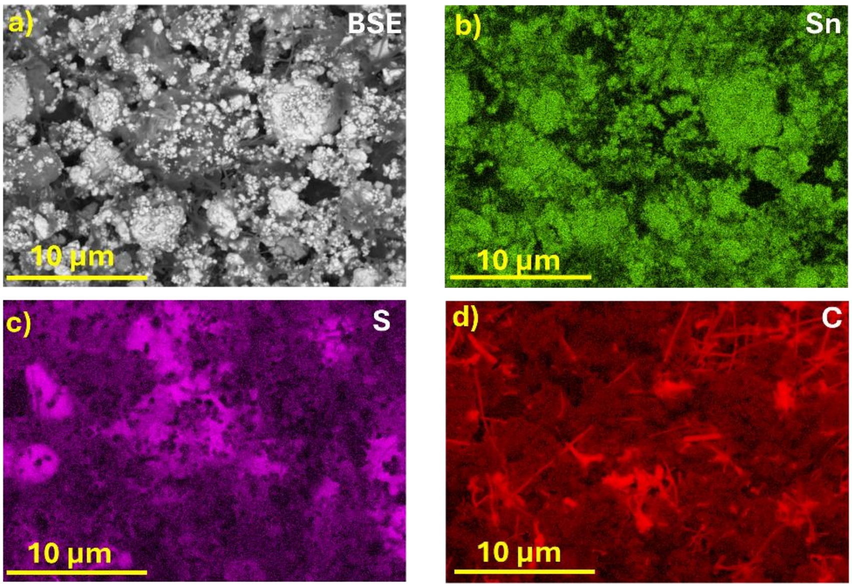

Figure S9: a) Back-scattered electron micrograph and elemental mapping for $Ni_{3.4}Sn_4$:Li_6PS_5Cl :VGCF / 78:20:2 $\%_{wt}$ with b) Sn (L-edge). c) S (K-edge) and d) C (K-edge).

[1] S. Furuseth, H. Fjellvag, Structural properties of $Ni_{3+x}Sn_4$, Acta Chem. Scand. A 40 (1986) 695–700.

[2] C. Suryanarayana, Mechanical alloying and milling, Progress in Materials Science 46 (2001) 1–184.

[3] S. Boulineau, M. Courty, J.-M. Tarascon, V. Viallet, Mechanochemical synthesis of Li-argyrodite Li_6PS_5X (X = Cl, Br, I) as sulfur-based solid electrolytes for all solid state batteries application, Solid State Ionics 221 (2012) 1–5.

[4] L. Peng, C. Yu, Z. Zhang, H. Ren, J. Zhang, Z. He, M. Yu, L. Zhang, S. Cheng, J. Xie, Chlorine-rich lithium argyrodite enabling solid-state batteries with capabilities of high voltage, high rate, low-temperature and ultralong cyclability, Chemical Engineering Journal 430 (2022) 132896.
